# Effects of steroids and angiotensin converting enzyme inhibition on circumferential strain in boys with Duchenne muscular dystrophy: a cross-sectional and longitudinal study utilizing cardiovascular magnetic resonance

**DOI:** 10.1186/1532-429X-13-60

**Published:** 2011-10-19

**Authors:** Kan N Hor, Wojciech Mazur, Michael D Taylor, Hussein R Al-Khalidi, Linda H Cripe, John L Jefferies, Subha V Raman, Eugene S Chung, Kathi J Kinnett, Katelyn Williams, William M Gottliebson, D Woodrow Benson

**Affiliations:** 1 The Heart Institute, Cincinnati Children's Hospital Medical Center, Cincinnati, Ohio, USA; 2 The Heart and Vascular Center at The Christ Hospitals, Cincinnati, Ohio, USA; 3 Duke University School of Medicine, Durham, North Carolina, USA; 4 The Ohio State University, Columbus, Ohio, USA

## Abstract

**Background:**

Steroid use has prolonged ambulation in Duchenne muscular dystrophy (DMD) and combined with advances in respiratory care overall management has improved such that cardiac manifestations have become the major cause of death. Unfortunately, there is no consensus for DMD-associated cardiac disease management. Our purpose was to assess effects of steroid use alone or in combination with angiotensin converting enzyme inhibitors (ACEI) or angiotension receptor blocker (ARB) on cardiovascular magnetic resonance (CMR) derived circumferential strain (ε_cc_).

**Methods:**

We used CMR to assess effects of corticosteroids alone (Group A) or in combination with ACEI or ARB (Group B) on heart rate (HR), left ventricular ejection fraction (LVEF), mass (LVM), end diastolic volume (LVEDV) and circumferential strain (ε_cc_) in a cohort of 171 DMD patients >5 years of age. Treatment decisions were made independently by physicians at both our institution and referral centers and not based on CMR results.

**Results:**

Patients in Group A (114 studies) were younger than those in Group B (92 studies)(10 ± 2.4 vs. 12.4 ± 3.2 years, p < 0.0001), but HR, LVEF, LVEDV and LVM were not different. Although ε_cc _magnitude was lower in Group B than Group A (-13.8 ± 1.9 vs. -12.8 ± 2.0, p = 0.0004), age correction using covariance analysis eliminated this effect. In a subset of patients who underwent serial CMR exams with an inter-study time of ~15 months, ε_cc _worsened regardless of treatment group.

**Conclusions:**

These results support the need for prospective clinical trials to identify more effective treatment regimens for DMD associated cardiac disease.

## Background

Duchenne muscular dystrophy (DMD) is an X-linked recessive disorder affecting approximately 1:3,500 males [1-3]. Boys with DMD are currently treated with corticosteroids at a young age to prolong ambulation. This therapy combined with improvements in respiratory care have resulted in increased survival [4-6] such that DMD-associated heart disease is now the leading cause of mortality [7-11]. Myocardial changes, as a result of the lack of dystrophin, consist of cell membrane degradation, interstitial inflammation, edema, fatty replacement and fibrosis [12-15]. Despite reports in small cohorts of the beneficial cardiovascular effects of corticosteroids [6,16] and angiotensin converting enzyme inhibitors (ACEI)/angiotensin receptor blockers (ARB) [17-19], there is no consensus regarding the management of DMD-associated cardiac disease. In the absence of conclusive randomized clinical trial data, steroids, ACEI, ARB, beta blockers (BB) and/or digoxin have been used empirically [20,21]; beneficial effects of positive inotropic agents have been reported in boys with depressed left ventricular function and advanced cardiac disease [22].

Defining clinical endpoints for cardiac therapy in DMD boys is challenging. Recent studies from our center and others have shown that indices of global left ventricular (LV) function, e.g. mass, volume and ejection fraction (EF) are not adequate to detect cardiac dysfunction in young DMD patients [23-25], but myocardial strain (ε), an indicator of local myocardial deformation normalized to its original dimension, can detect occult cardiac disease early in the course of DMD despite normal EF. Further, depressed ε_cc _magnitude correlates with disease progression [24,25].

The purpose of this study was to retrospectively compare cardiac effects of corticosteroid monotherapy versus corticosteroids plus ACEI or ARB in a cohort of DMD patients followed at our center. We compared effects on both global LV function (EF) and local LV function (**ε**_cc_) determined by cardiovascular magnetic resonance (CMR).

## Methods

DMD patients >5 years of age who underwent CMR from February 2006 to February 2010 were identified from the CMR database at Cincinnati Children's Hospital Medical Center (CCHMC). The diagnosis of DMD was confirmed by physical examination and identification of a dystrophin mutation. This study was approved by the Institutional Review Board.

Patients were identified for inclusion in one of two treatment groups. Group A was only treated with corticosteroids (either deflazacort or prednisone). Group B was being treated with corticosteroids plus ACEI (lisinopril or enalapril) or ARB (losartan). All patients in Group B had been treated with ACEI/ARB for a minimum of 12 months prior to CMR, and all patients in both groups had been treated with corticosteroids for at least 12 months prior to CMR. Initiation of corticosteroids and ACEI/ARB was determined exclusively by treating physician preference and were not based on CMR results. DMD boys not treated with corticosteroids or treated with beta blockers were excluded.

CMR studies were conducted on either a 3 Tesla (Trio, Siemens Medical Solutions, Erlangen, Germany) or 1.5 Tesla (Signa Excite, General Electric Healthcare, Milwaukee, WI) scanner based on clinical availability, independent of the patient's clinical status or prior type or field strength of the first study. Cardiac functional imaging was performed using retrospective ECG-gating, segmented Steady State Free Precession (SSFP) technique after localized shimming and/or frequency adjusting. Subjects were breath-held as tolerated; for those subjects who could not adequately breath-hold, a free breathing technique with multiple signal averaging was used. Standard imaging included a short axis stack of cine SSFP images from cardiac base to apex; the short axis was prescribed as the perpendicular plane to the left ventricular long axis in 2 and 4 chamber views as previously described [26,27]. Typical scan parameters included FOV = 32-38 cm, slice thickness = 5-6 mm, gap = 1-2 mm, NEX = 2 (breath hold; 4-5 for free breathing), TE/TR = 1.4/2.8 (Siemens), TE/TR = 2.0/4.0 (GE), in-plane resolution = 1.2- 2.2 mm. A minimum of 12 slices were performed, with 20 phases/slice. The typical temporal resolution of the cine SSFP images was 30-40 ms; they and were adjusted according to the patient heart rate and ability to breath-hold. The RF flip angles were set between 50°-70° dependent on the patient weight, height and the SAR level.

Tagged cine CMR images were acquired in the short axis of the midventricle at the level of the papillary muscles using an ECG-triggered segmented k-space fast gradient echo sequence with spatial modulation of magnetization in orthogonal planes. Tag imaging was performed prior to administration of Gadolinium. Grid tag spacing was 7-8 mm. The scan parameters used were: field of view = (30-32) × (25-26) cm^2^, slice thickness = 6 mm, flip = 20°, TE/TR = 3 ms/6.6 ms (GE), = 3 ms/4.2 ms (Siemens), views per segment = 8 (GE), = 7-9 (Siemens). Tagged images were analyzed using the HARmonic Phase (HARP, Diagnosoft, CA, USA) technique [23,28-32]. Only the mid-ventricular slice was analyzed, based on our experience and others' [23] of limited reproducibility of the basal and apical slices. Details of the ε_cc _analysis have been previously described by Hor et al. [24]. The ε_cc _data was exported to a spreadsheet file for analysis.

Cardiac functional imaging was performed to determine LV volumes, mass and EF using semi-automated endocardial and epicardial segmentation (QMASS v.6.1.5, Medis Medical Imaging Systems) Netherlands and circumferential strain (**ε**_cc_) using HARmonic Phase analysis (HARP, Diagnosoft, Palo Alto, California) as previously described [23-25,29-31,33-35].

Results are expressed as mean ± SD for continuous data and as percentages and numbers for categorical data. Continuous variables were compared using two-sample t-test and categorical variables were compared using Fisher exact-test. Analysis of covariance (ANCOVA) was used to assess the effect of medications on several clinical measurements with age as a continuous covariate in the model. All tests were 2-sided, and a p-value < 0.05 was considered statistically significant. SAS version 9.2 (SAS Institute Inc., Cary, NC) was used for all analyses.

## Results

We identified 171 DMD patients > 5 years of age that had undergone CMR examination. A total of 256 studies were available for review as a number of patients had multiple studies. We excluded 50 studies for either no corticosteroid treatment at the time of study (36 studies) or treatment with beta-blockers at the time of study (14 studies). The remaining 206 studies from 136 patients were divided into two groups: Group A (corticosteroids alone, n = 114) and Group B (corticosteroids plus ACEI or ARB, n = 92). Group A patients were significantly (p < 0.0001) younger (10 ± 2.4 years) than Group B patients (12.4 ± 3.2 years).

The average steroid dose was similar between Group A and Group B (0.7 ± 0.29 mg/kg/day, range 0.1-1.9 vs. 0.6 ± 0.22 mg/kg/day, range 0.1 - 1.3, p = 0.48). The patients in Group B were on standard dose of ACEI (0.16 ± 0.29 mg/kg/day, range 0.04 - 1.23) and ARB (0.73 ± 0.29 mg/kg/day, range 0.34 - 1.23) (Table 1).

**Table 1:** DMD Patients characteristics

Parameter	**Steroid Only (A)(n = 114 )**	**Steroid plus ACEI_ARB (B)(n = 92 )**	P-value
Age (yrs)	10.0 ± 2.4	12.4 ± 3.2	<0.0001
Heart Rate (bpm)	101 ± 19	104 ± 15	0.2498
LVEDV (mL)	82.5 ± 21.8	86.7 ± 24.8	0.2023
LVM (g)	58.6 ± 19.4	62.1 ± 19.8	0.1031
EF (%)	64.2 ± 6.1	62.8 ± 7.5	0.1414
ε_cc _(%)	-13.8 ± 1.9	-12.8 ± 2.0	0.0004
Steroid dose (gram/kg/day)	0.7 ± 0.29	0.6 ± 0.22	0.4838
ACE-I dose (gram/kg/day)	N/A	0.16 ± 0.08	N/A
ARB dose (gram/kg/day)	N/A	0.73 ± 0.29	N/A

Abbreviations: ACE-I = Angiontension Converting Enzyme Inhibitor, ARB = Angiotension Receptor Blocker, bpm = beat per minute, Clinic Prior to CMR = Previous Clinic Visit Documenting Medication and Dose Prior to Cardiac Magnetic Resonance Imaging Study, DMD = Duchenne Muscular Dystrophy, ε_cc _= Circumferential Strain, EF = Ejection Fraction, LVEDV = Left Ventricular Endiastolic Volume, LVM = Left Ventricular Mass.

Heart rate, LV EF, LV end diastolic volume (LVEDV) and LV mass (LVM) were not different between the two groups. LVEF averaged across all patients in this cohort was normal, but **ε**_cc _magnitude was significantly lower in Group B (-12.8 ± 2) versus Group A patients (-13.8 ± 1.9, p = 0.0004). To determine the extent to which differences in the two groups was an effect of patient age, we performed a covariance analysis and treated age as a continuous variable (Table 2). The covariance model confirmed the lack of significant differences between Groups A and B with respect to heart rate, EF, LVEDV, LVM, LVRI or **ε**_cc _after controlling for age, implying lack of difference between the two groups other than the use of cardiac medications in Group B.

**Table 2:** Analysis of covariance summary results: Comparisons between steroid only vs. steroid plus ACEI_ARB (medication) adjusted for age as a continuous variable

Response Variable	Medication		Age	
	
	F-statistics	P-value	F-statistics	P-value
Heart Rate (bpm)	1.11	0.2930	0.00	0.9979
LVEDV (mL)	1.33	0.2503	37.01	<0.0001
LVM (g)	2.19	0.1405	64.38	<0.0001
EF (%)	0.03	0.8594	9.10	0.0029
ε_cc _(%)	1.74	0.1885	30.85	<0.0001

Abbreviations: ACE-I = Angiontension Converting Enzyme Inhibitor, ARB = Angiotension Receptor Blocker, bpm = beat per minute,?ε_cc _= Circumferential Strain, EF = Ejection Fraction, LVEDV = Left Ventricular Endiastolic Volume, LVM = Left Ventricular Mass.

**Figure 1:**
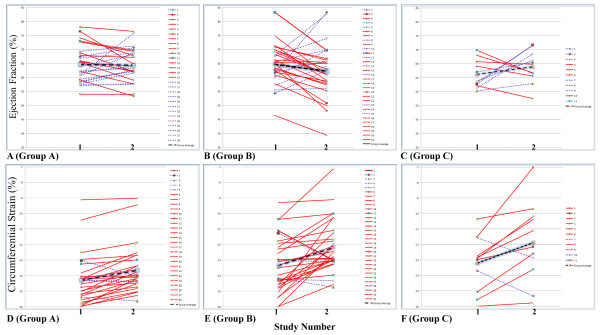
**Serial ejection fraction (EF) and circumferential strain (ε_cc_)**. *Red*, indicates decreased EF and ε_cc _magnitude; *blue*, indicates increase EF and ε_cc _magnitude and *black*, indicates mean EF and ε_cc _values. Panels A-C show EF of each subject plotted serially; there was no significant change in mean EF across all three groups. Individually 14/28 (50%) in Group A, 21/31(68%) in Group B and 5/11 (45%) in Group A to B transition had decrease EF, while 14/28 (50%), 10/31 (32%) and 6/11 (55%) of the individuals in the respective groups had increase EF. Panels D-F show ε_cc _of each subject plotted serially; although all three groups had lower absolute ε_cc _on the follow-up study, only Group B subjects had a significant decline (p = 0.007). Individually ε_cc _magnitude decreased more consistently across all three groups, with 24/28 (86%) in Group A, 26/31 (84%) in Group B and 9/11 (82%) in Group A to B. Unlike EF, only a small fraction of the individuals have improvement of ε_cc _magnitude on follow-up study with 4/28 (14%), 5/31 (31%) and 2/11 (18%) of the individuals in the respective group having an increase ε_cc _value on follow-up study.

Additionally, to assess serial changes in EF and **ε**_cc _we identified patients in Group A (n = 28) and B (n = 31) who underwent 2 CMR studies during the observation period. This included 11 patients who crossed from Group A to B between sequential studies. Among individuals with 2 studies, EF and **ε**_cc _values were compared between the two studies (Figure 1). EF changes between the first and second study were variable across three groups, while the majority of patients had decline in **ε**_cc _magnitude over the same study period. In Group A, 50% (14/28) patients had decrease EF while the other 50% (14/28) had increase EF. Of Group A patients, 7% (2/28) had changes in EF by > 10%, however one increased by almost 15% and the other decrease by 12%. Both patient s continue to have normal EF (>55%) on both studies. One patient had EF < 55% on the first study which remained the same on the second study and another patient had EF that decline from 59% to 54%. However, only 14% (4/28) of Group A subjects had an increase **ε**_cc _magnitude while 86% (24/28) had worsening of **ε**_cc _magnitude. The findings for Group B were similar, with 68% (21/31) of patients having decrease EF and 32% (10/31) of patients with increase EF over the two study. Of Group B patients, 28% (9/32) had changes in EF by > 10% with 6 patients having decrease EF and 3 patients with increase EF on the follow-up study. Of these, One patient started with an EF of 53% and increased to 64% on the follow-up study. Of the 6 patients with decrease in EF, three had EF < 55% while the other three continue to have normal EF on the follow-up study. As in Group A, 84% (26/31) of the Group B patients had decrease **ε**_cc_, while only 16% (5/31) subjects showed improvement in **ε**_cc _magnitude. For the patients that crossed from Group A to B, the percent of patients with improved EF was 45% (5/11) while 55% (6/11) had decline in EF. The percent of patients with an increase in **ε**_cc _magnitude was 18% (2/11) but 82% (9/11) had worsening of **ε**_cc _magnitude (Figure 1). The average time between studies was the same across Group A, Group B and patients who transitioned from Group A to B. LVEF did not change significantly between baseline and follow-up in all three groups (Table 3). While **ε**_cc _worsened from initial to follow-up CMR exam in all three groups, reduction in **ε**_cc _only reached statistical significance in Group B (Table 3).

**Table 3:** Results from Serial CMR Exams Across DMD Treatment Groups

Group/Patients Time Interval (months)	Group A (Steroids); n = 28 Mean = 14.9 ± 5.6 Range = 8.5-29.7			Group B (Steroids plus ACEI/ARB); n = 31 Mean = 15.1 ± 5.9 Range = 8.4-35.8			Group A to B; n = 11 Mean = 15.6 ± 5.9 Range = 5.8-25.5		
CMR Study	Study 1	Study 2	P-value	Study 1	Study 2	P-value	Study 1	Study 2	P-value
Age (yrs)	9.30 ± 1.5	10.5 ± 1.6	<0.005	11.7 ± 3.4	12.97 ± 3.4	0.148	10.8 ± 2.5	12.0 ± 2.2	0.252
HR (bpm)	101 ± 21	99 ± 16	0.762	105 ± 14	105 ± 15	0.996	100 ± 14	102 ± 18	0.682
LVEDV (mL)	82.7 ± 19.3	86.5 ± 21.6	0.494	84.9 ± 29.2	90.0 ± 30.8	0.499	85.6 ± 18.6	81.2 ± 15.6	0.556
LVM (g)	57.1 ± 15.1	57.7 ± 16.9	0.890	60.9 ± 21.4	65.0 ± 21.4	0.478	61.3 ± 31.2	60.1 ± 14.0	0.909
EF (%)	64.6 ± 6.3	64.4 ± 5.8	0.906	64.9 ± 6.7	62.2 ± 9.1	0.194	61.2 ± 5.0	63.8 ± 5.8	0.261
ε_cc _(%)	-14.3 ± 1.6	-13.7 ± 1.5	0.135	-13.4 ± 1.7	-12.1 ± 1.6	0.007	-13.2 ± 1.8	-11.9 ± 2.7	0.179

Abbreviations: BMP = beat per minute, DMD = Duchenne Muscular Dystrophy, ε_cc _= Circumferential Strain, EF = Ejection Fraction, ACEI = angiotensin converting enzyme inhibitor, ARB = angiotensin receptor blocker, HR = heart rate, LVEDV = Left Ventricular Endiastolic Volume (milliliter), LVM = Left Ventricular Mass (gram).

## Discussion

This study represents the largest cohort of DMD patients analyzed for the effect of medical therapy on the natural history of DMD-associated cardiac disease. Albeit retrospective, these results suggest that treatment with either an ACEI or ARB does not arrest the decline in DMD-associated cardiac function in patients receiving corticosteroids. In fact, we found a statistically significant worsening in **ε**_cc _in patients taking both corticosteroids and ACEI or ARB, suggesting that current approaches are inadequate in arresting decline in cardiac function.

Clinical assessment of DMD-associated cardiac disease is particularly challenging, as overt decompensation typically occurs only in the terminal stages of disease at a time when clinical assessment of cardiac function is limited. A sensitive and specific parameter such as **ε**_cc _could thus assume great importance in management of these patients. Previous studies have shown that changes in **ε**_cc _precede decline in LVEF in DMD-associated cardiac disease as well as in both hypertrophic and hypertensive cardiomyopathy [24,36-38]. Using such an early marker of subclinical disease in a cohort with preserved LVEF, we were unable to detect an improvement with standard therapies.

Assessment of cardiac effects of corticosteroid therapy in animal models and patients has historically yielded mixed results. For example, corticosteroids have been shown to improve systolic function in children with myocarditis but not with dilated cardiomyopathy [39]. Markham et al demonstrated that freedom from ventricular dysfunction at approximately 50 months between retrospectively-reviewed echocardiograms was 93% for steroid treated versus 53% for untreated DMD cases [6]. However, in a mouse model of dystrophin deficiency, Bauer et al demonstrated acceleration of cardiac dysfunction in steroid treated animals [40]. Recently, Pereira et al [41] observed normalization of multidirectional (radial, longitudinal and circumferential) strain assessed by 2D echo speckle tracking in Cushing syndrome patients following normalization of corticosteroid excess; they concluded that corticosteroid excess not only induced LV hypertrophy and diastolic dysfunction but also subclinical systolic dysfunction, which reverses upon normalization of corticosteroid excess. While there is universal agreement that steroids prolong ambulation in the DMD patient population (reviewed in [21]), the current study failed to identify a cardioprotective effect of steroids.

Several studies have suggested beneficial effects of ACEI on LV function in small cohorts [17-19,42]. In a murine DMD model, Bauer et al showed hemodynamic benefit to dystrophin-deficient mice treated with ACEI alone [40]. Jefferies et al suggested that this effect may be linked to specific mutations [19] in humans, but Ramaciotti et al [42] did not confirm this finding. In addition to **ε**_cc_, we found no difference in EF between baseline and 15 months, which is at odds with the findings of Duboc et al [17] who found that treatment with perindopril delayed the onset and progression of LV dysfunction in children. There are several possible explanations for the differences in findings. First, in our cohort, the time between the serial CMR studies might be insufficient to develop detectable LV EF decline. However, one would expect to detect more subclinical changes using the more sensitive measure LV **ε**_cc_. Furthermore, without an untreated control group, the fact that **ε**_cc _magnitude declined only slightly may represent a "good" outcome, as LV function of untreated patients may decline more precipitously. Despite smaller studies reporting benefit of ACEI and BB, there is no published data demonstrating longer life expectancy in DMD boys undergoing "adult-like" CHF therapy.

A long-standing hypothesis regarding DMD-associated cardiac disease pathogenesis is that loss of membrane integrity is a primary event leading to myocyte degeneration. Intermittent tears in the cell membrane permit influx of calcium that then functions as a primary inducer of a destructive cascade culminating in myocyte necrosis and replacement fibrosis [43]. Although there has been intense interest in treating DMD-associated cardiac disease, successful therapies remain elusive [22,44-46]. Findings in the current study underscore the need for randomized studies in DMD population. As in cases of heart failure in adult population, development of heart failure with normal EF starts early (HFNEF) and proceeds to systolic dysfunction. Of interest, studies in adult HFNEF patients treated with ACEI or BB have not demonstrated improvement in mortality compared to placebo [47-49]. In boys with DMD, dystrophin mutation dictates eventual LV dysfunction and once systolic dysfunction and myocardial fibrosis develop, clinical disease typically progresses very rapidly and long-term survival (as seen in adult patients with LV systolic dysfunction) is poor. As such, the window of opportunity to treat DMD-associated cardiac disease needs to be advanced to when **ε**_cc _is abnormal but EF has not yet begun its inexorable declined.

### Study Limitations

Study design based on physician preference is a major limitation of the study and includes retrospective, mostly cross-sectional data with no control group and with strong confounding of group allocation by age of boys being prescribed therapy. Older boys tended to be on combination treatment while younger boys were often treated with steroids alone. Further, longitudinal data is limited. Other confounding factors are very difficult to control for but are beyond the scope of this manuscript. As such, no firm conclusions can be drawn regarding the response rates to steroid or ACEI/ARB use. Further, findings are limited to patients >5 years since in our experience patients < 5 years of age cannot undergo CMR without sedation. While it is possible, that 15 months follow-up was too short to detect therapeutic benefits, studies in other populations with preserved EF but measurable diastolic dysfunction have shown improvement in circumferential and longitudinal strain 12 months after ARB initiation [50].

## Conclusions

These results support the need for rigorous, prospective clinical trials to identify more effective treatment regimens for DMD-associated cardiac disease.

## List of Abbreviations

ACEI: Angiotensin Converting Enzyme Inhibitor; ARB: Angiotension Receptor Blocker; BB: Beta blockers; bpm: beat per minute; Clinic Prior to CMR: Previous Clinic Visit Documenting Medication and Dose Prior to CMR; CMR: Cardiovascular Magnetic Resonance; DMD: Duchenne Muscular Dystrophy; ε_cc_: Circumferential Strain; EF: Ejection Fraction; Group A: Steroid only; Group B: Steroid plus; HR: heart rate; LV: Left Ventricular; LVEDV: Left Ventricular Endiastolic Volume; LVM: Left Ventricular Mass; MO: months.

## Competing interests

The authors declare that they have no competing interests.

## Authors' contributions

KNH and DWB contributed to all aspects of the manuscript's conception, design, data analysis, collection, critical revision and final approval. HRA, contributed to statistical analysis, revision and final approval. MDT, LHC, JLJ, SVR, ESC, KJH, KW and WMG* contributed in interpretation of the data, critical revision and final approval of the manuscript. All authors have read and approved the final manuscript.

*WMG passed on September 17, 2010 and therefore, did not have the opportunity to give final approval of the manuscript but contributed significantly to it.
